# Spray of hydrogen peroxide for infection prevention and control of SARS COV 2 infection: could this be possible?

**DOI:** 10.11604/pamj.supp.2020.35.2.23284

**Published:** 2020-06-09

**Authors:** Cinzia Casu

**Affiliations:** 1 DDS, Private Dental Practice, Cagliari, Italy

**Keywords:** Hydrogen peroxide, COVID 19, spray against SARS COV 2

## To the Editors of the Panafrican Medical Journal

The COVID 19 pandemic caused from SARS CoV 2 was declared on March 11, 2020 [1]. SARS-CoV2 belongs from the beta Coronavirus family, it is a single-stranded RNA, enveloped virus that is 50-200 nm in diameter [1]. According to a recent revision, the world mortality rate is 12%. The number of scientific publications that has been produced in less than 4 months is very high: typing the keyword “coronavirus COVID 19” appear over 4700 results on the PubMed database. It seems that scientific research is directed more towards finding a drug for the treatment of COVID 19, rather than preventing the spread of the virus. As mentioned in January by a group of Chinese researchers [2] oxidizing solutions such as hydrogen peroxide could be effective in the oral cavity. However, when I typed the keywords “coronavirus hydrogen peroxide” on PubMed, I obtained only 13 results and with “coronavirus COVID 19 hydrogen peroxide” I found 4 results (May, 4, 2020). Hydrogen peroxide, a molecule that consists of hydrogen and oxygen (H2O2), was discovered for the firts time, as a biocidal agent from the early 1800s by chemist Louis-Jacques Thenard (1777-1857). The work of early pioneers in disinfection, such as Benjamin Ward Richardson (1828-96), enhanced the applications of H2O2. Today, its use as a disinfectant is very increased, also as general surface disinfectant in dental field [3]. Hydrogen peroxide (HP) in its pure form is light blue, in its diluted form is a colorless, odorless watersoluble liquid. When concentrated, acts as a strong oxidizing agent [4]. It is used in surgery because it has an antispetic effect, improves hemostasis and wound healing, re-epithelialization, and reduce inflammation [4]. A HP concentration comprised between 3% to 6% is bactericidal, and slowly sporicidal. Higher concentrations comprised between 10% to 30% showed much better sporicidal activity in vitro [4]. Its toxicity is very low, in fact authors reported that ingestion of 3% hydrogen peroxide may cause gastrointestinal irritation and whitening of the mucosa, but these are mostly benign collateral events [4].

The use of HI also against virus infection is well documented in the scientific literature. Goyal et al. studied the effectiveness of a vapour of HI versus different viruses: feline calicivirus; human adenovirus type 1; transmissible gastroenteritis coronavirus of pigs (very similar to SARS-CoV); avian influenza virus (AIV); and swine influenza virus (SwIV). Authors demonstrated that adenovirus, TGEV and AIV died at the lowest vaporized HI volume tested (25 ml). They evaluated that the total exposure time, including injection and aeration, was 2-3 hours, varying with the amount of hydrogen peroxide being vaporized [5]. Holtkamp et al. in an animal study, treated pigs infected with porcine epidemic diarrhea virus (PEDV) with a 1:16 or 1:32 concentration of HI disinfectant. The contact time was 30 min at 20 degrees. They obtained a good result [6]. Rudnick et al. proposed an HI vapour to disinfect surface with H1N1 influenza virus. These virus was deposited in aqueous suspensions on stainless-steel surface at ambient conditions, and then exposed for up to 15 minutes to different concentration between 10 to 90 ppmof HP vapor. After 2.5 minutes, a minimal exposure to 10-ppm HP vapor inactivated 99% of H1N1 virus [7]. Lee et al. showed that HI could be spontaneously produced from pure water through a process of atomizing bulk water into droplets comprised between 1 μm to 20 μm in diameter [8]. Water is a stable and relatively inert molecule in bulk solution. The water have an exceptional behavior: they found in this in vitro work, that water molecules are spontaneously oxidized to form hydrogen peroxide near the water-air interface of micron-sized water droplets. A process that does not require any chemical reagent, catalyst, applied electric potential, or radiation [8].

An hydrogen peroxide spray for decontamination of surfaces in hospitals was successfully tested by Cadnum et al. They observed that a 1.4% solution of hydrogen peroxide (IHP) spray disinfectant could be very effective against Staphilococcus aureus and strains of vancomycin-resistant enterococci [9]. In a very recent review of January 2020 researchers found that the analysis of 22 studies reveals that human coronaviruses such as Severe Acute Respiratory Syndrome (SARS) coronavirus, Middle East Respiratory Syndrome (MERS) coronavirus or endemic human coronaviruses (HCoV) could be inactived by 0.5% hydrogen peroxide solution. Coronavirus can persist on inanimate surfaces like metal, glass or plastic for up to 9 days. Disinfection procedures with 62-71% ethanol, 0.1% sodium hypochlorite or 0,5% HI could be effective to prevent the spread [10]. Could hydrogen peroxide spray solutions, which can be scarcely cytotoxic if inhaled, with extremely low costs, be the subject of clinical studies and usable for the prevention of the spread of SARS CoV 2? I hope this consideration of mine can be a starting point for future research.

## Competing interests

The authors declare no competing interests.
